# Implementation of Antimicrobial Stewardship in the Healthcare Setting

**DOI:** 10.7759/cureus.26664

**Published:** 2022-07-08

**Authors:** Nandkishor J Bankar, Sarita Ugemuge, Ranjit S Ambad, Dattu V Hawale, Dilip R Timilsina

**Affiliations:** 1 Microbiology, Jawaharlal Nehru Medical College, Datta Meghe Institute of Medical Sciences, Wardha, IND; 2 Microbiology, Datta Meghe Medical College, Datta Meghe Institute of Medical Sciences, Nagpur, IND; 3 Biochemistry, Datta Meghe Medical College, Datta Meghe Institute of Medical Sciences, Nagpur, IND; 4 Biochemistry, Jawaharlal Nehru Medical College, Datta Meghe Institute of Medical Sciences, Wardha, IND

**Keywords:** antimicrobial resistance, health care, policy, hospital infection control, antimicrobial stewardship

## Abstract

Antimicrobial resistance (AMR) is a serious problem that poses an imminent threat to patient safety. But drug-resistant bacteria can be prevented from spreading in hospital facilities by implementing effective antimicrobial stewardship practices. Antimicrobial stewardship programs are a set of measures taken by an organization to optimize antimicrobial use, improve patient outcomes, reduce AMR and healthcare-associated infections, and save healthcare costs. Healthcare facilities should have a defined antimicrobial stewardship policy in place that is available to all stakeholders. The policy should be evidence-based, regularly updated, and communicated clearly both verbally and through visual means such as posters. All staff should be trained on the proper use of antimicrobials as well as how to report misuse. Antibiotic stewardship measures include: educating and screening patients, monitoring, updating policies, limiting the use of high-risk medications, developing and improving hand hygiene practices, tracing the path of each medication, using computerized alert probes, using computerized medication records, educating staff, and creating the culture of prevention. There are several ways that antimicrobial stewardship practices can be implemented in the healthcare setting, including limiting the use of antibiotics and promoting healthy behaviors. With these strategies in place, infections can be prevented from occurring in the first place.

## Introduction and background

The prevalence of drug-resistant bacteria is increasing in hospitals and other healthcare settings. Several studies have found that adverse medication events, healthcare-associated infections (HCAIs), and surgical complications are the most common types of adverse events impacting hospitalised patients. According to the US Centers for Disease Control and Prevention, about 1.7 million hospitalised patients get HCAIs each year while being treated for other health concerns, and more than 98,000 individuals (one in 17) die as a result of hospital-acquired infection [1]. Antimicrobial resistance is a serious problem that poses an imminent threat to patient safety. But drug-resistant bacteria can be prevented from spreading in hospital facilities by implementing effective antimicrobial stewardship practices [2].

Antimicrobial resistance (AMR) is an emerging global public health crisis that threatens to undermine the last century’s advances in infectious disease control and threaten the lives of millions worldwide unless action is taken now. AMR is the global health threat caused by the overuse and misuse of antibiotics, as well as by the emergence of drug-resistant bacteria. The problem is exacerbated by poor infection control practices in hospitals, long drug-supply chains, inadequate regulation of antibiotic sales, and poor access to clean water and sanitation in many parts of the world. The world is running out of effective antibiotics because of the increase in resistance to antibiotics. Global Antimicrobial Resistance and Use Surveillance System (GLASS) reported that ciprofloxacin resistance varies from 8.4%-92.9% for *Escherichia coli *and 4.1%-79.4% for *Klebsiella pneumoniae*. Worldwide *Klebsiella pneumoniae *has developed resistance to the carbapenems used as a last option. Additionally, colistin-resistant bacteria have been found in a number of nations and locations, where they are producing diseases for which there is now no effective antibiotic treatment [3].

The World Health Assembly’s endorsement of the Global Action Plan on Antimicrobial Resistance in May 2015 was a critical milestone in the global effort to tackle AMR. The plan has five objectives:

1. To improve awareness and understanding of antimicrobial resistance through effective communication, education, and training;

2. To strengthen the knowledge and evidence base through surveillance and research;

3. To reduce the incidence of infection through effective sanitation, hygiene, and infection prevention measures;

4. To optimize the use of antimicrobial medicines in human and animal health; and

5. To develop the economic case for sustainable investment that takes account of the needs of all countries and to increase investment in new medicines, diagnostic tools, vaccines, and other interventions.

This plan was taken forward by a series of calls to action from WHO Director-General Dr. Tedros Adhanom Ghebreyesus and other international organizations, including the United Nations International Children's Emergency Fund (UNICEF) and the United Nations Office on Drugs and Crime (UNODC). It also provided a platform for more than 200 countries, including many developing countries, to pledge their support for actions to address AMR [4].

Antimicrobial stewardship programs are a set of measures taken by an organization to optimize antimicrobial use, improve patient outcomes, reduce AMR and healthcare-associated infections, and save healthcare costs. These programs aim to ensure that all healthcare workers follow guidelines on the appropriate use of antimicrobial drugs, among others. They aim to reduce the emergence and spread of resistance by ensuring that all health care workers follow appropriate protocols for the use of antimicrobials (e.g., proper dosage, duration of treatment, and the proper use of diagnostic tests).

In this review, we will discuss what exactly antimicrobial stewardship is and why it’s so essential for a healthcare organization. Also, it focuses on key strategies for implementing antimicrobial stewardship programs in the organization - wherever it may be located.

## Review

What is antimicrobial stewardship?

Antimicrobial stewardship is "the optimal selection, dosage, and duration of antimicrobial treatment that results in the best clinical outcome for the treatment or prevention of infection, with low harm to the patient and minimal impact on eventual resistance” [5].

Antimicrobial stewardship is a strategy for limiting the use of antibiotics only when and where needed [6]. Antibiotics are powerful drugs that kill bacteria but don’t discriminate between pathogens and commensal. That means that when antibiotics are used, they can kill both the pathogenic bacteria and the commensal bacteria in the body. This can result in adverse outcomes of antibiotic resistance [7]. Stewardship has been used to govern the health sector as a whole, assuming responsibility for the population's health and well-being and steering health systems at the national and global levels [4].

The goal of antimicrobial stewardship can be:

1. To collaborate with healthcare providers to ensure that each patient receives the most suitable antibiotic at the right dose and for the right length of time by following the four D’s: “right Drug, right Dose, De-escalation to pathogen-directed therapy, and right Duration of therapy” [8]

2. To avoid the overuse, misuse, and abuse of antimicrobials in the hospital as well as in the outpatient setting

3. To prevent the emergence and development of resistance at the patient level and community level as discriminated antibiotic use can change the susceptibility pattern

Why is antimicrobial stewardship important?

The overuse of antibiotics can create several problems. For one thing, it can increase the spread of antibiotic resistance. But it can also directly impact the health of patients and those in healthcare [9].

Here’s why antimicrobial stewardship is so important. Overuse of antibiotics is responsible for antimicrobial resistance and may lead to higher medical costs and increased mortality because they do not treat the infections effectively. A good example is *Clostridioides difficile *infection (CDI), a serious bacterial infection that can occur in patients who have taken an antibiotic (either recently or in the past) and whose immune systems have been weakened by illness or surgery. CDI is frequently treated with high doses of vancomycin - but if that treatment is not given early enough in the course of the illness, CDI symptoms will often return when people stop taking vancomycin. In addition, CDI symptoms are frequently more severe than those experienced by people who get CDI without ever having taken an antibiotic for it. When this happens, patients are at risk for additional complications from their disease and treatment. Prescribing antibiotics only when they’re truly necessary sets up a situation where fewer people develop CDI complications in the first place, and those who do develop them respond better to appropriate treatment with antibiotics [10].

Antimicrobial stewardship measures

Educating and Screening Patients

Communicating with patients and screening them before they are prescribed antibiotics can help prevent unnecessary antibiotic use in healthcare settings. Educating and screening patients can determine whether antibiotics are truly needed in a particular case. The Centers for Disease Control and Prevention have published a valuable checklist for screening patients for antibiotics to help healthcare facilities implement this practice more effectively [11]. Some common reasons for not prescribing antibiotics include: - Patients’ symptoms do not meet the clinical criteria for bacterial infection - The patient has a history of allergies to antibiotics - The patient has an alternative treatment that is likely to be effective - The patient is likely to be allergic to the antibiotic being prescribed - The patient has another condition that could affect his or her response to the antibiotic, or a drug that could adversely interact with the antibiotics [12].

Monitoring

By monitoring prescribing practices and conducting interventions in programs like Plan-Do-Study-Act (PDSA), hospitals can help reduce preventable antibiotic-resistant infections and eradicate the threat of untreatable infections. Monitoring prescribing patterns in the organization may be able to reduce inappropriate antibiotic use. When this happens, reductions in resistance rates and better therapeutic outcomes can be seen. Monitoring these outcomes is an important part of your overall strategy. If these are not monitored, it is difficult to know if efforts are successful or not [13].

Updating Policies

One of the best ways to improve patient care and minimize unnecessary use of medications is by updating hospital policies pertaining to antimicrobial stewardship campaigns within your facility. In addition to monitoring patient outcomes after implementing a program for antimicrobial stewardship in your hospital, it is important to monitor changing susceptibility trends among healthcare facilities and obtain national benchmarks from other hospitals with similar populations as yours for comparison purposes, which can determine whether or not healthcare facilities are making improvements over time. It is important to gather background data on current antibiotic prescription and use rates [14]. The cumulative antibiogram of diverse organisms isolated from patients can be created to track geographic trends of resistance utilising information from multiple facilities [15].

Limiting the Use of High-Risk Medications

Certain types of antibiotics are more likely to encourage the development of drug-resistant bacteria than others. Limiting the use of these high-risk antibiotics in a healthcare setting can help reduce the risk of antimicrobial resistance [16]. The CDC has created a list of high-priority antibiotics to help determine which medications should be used only when absolutely necessary. In addition, they’ve created a list of low-priority antibiotics that should be used only in extreme cases. In addition to limiting the use of high-risk antibiotics, we can also combat antimicrobial resistance by expanding the use of low-risk antibiotics. By doing so, it is ensured that antibiotics are only used when absolutely necessary [11]. The WHO Expert Committee on Selection and Use of Essential Medicines developed the AWaRe Classification of antibiotics in 2017 to support antibiotic stewardship efforts at local, national, and global levels. It categorizes antibiotics into three groups - Access, Watch, and Reserve - taking into account the consequences of different antibiotics and antibiotic groups on antimicrobial resistance. As a result, the importance of their appropriate usage is emphasised [17].

Developing and Improving Hand-Hygiene Practices

Hand hygiene is one of the most effective ways to prevent the transmission of infections from one patient to another. It is particularly important in settings where patients are receiving intravenous therapy [18]. If certain bacteria are resistant to antibiotics, you can reduce the instances of infection by simply washing your hands properly. The CDC has a list of different steps that healthcare workers should follow to ensure proper hand hygiene: Make sure that all healthcare providers have access to hand hygiene supplies at all times. Antibacterial soap or 2% chlorhexidine gluconate solution is a good option for proper hand hygiene. However, any alcohol-based hand rub will work just as well. Facilities should also have alcohol-based hand rubbing dispensers installed throughout the healthcare setting for easy access by all staff members. Furthermore, instruments such as continued education, bedside observation, and the introduction of innovative tools, such as electronic wearables and Wi-Fi-enabled dispensers, are all choices that can help improve the present low levels of hand hygiene compliance [19].

Tracing the Path of Each Medication

Every time a medication is used in a healthcare setting, there is a risk that errors may occur. That is why it is important to trace the path of every medication from the moment it is first prescribed to the moment it is administered to the patient. In order to trace the path of each medication, it is important to keep a record of medication orders and administration. For each medication, create a medication administration record that documents the date it was issued, the name of the patient it was prescribed for, and the name of the healthcare provider who prescribed it. An organization should also document the time and location of administration on this record. Tracing each medication’s path can help you identify potential problems, including whether a particular medication has been used on multiple patients [20]. These approaches also notify prescribers when there is an unnecessary overlap in antimicrobial coverage, and they aid in the detection and prevention of antibiotic medication interactions [21].

Using Computerized Alerts and Probes

Keeping track of the use of each medication can be challenging. But by using computerized alerts and probes to monitor the use of medications, you can reduce the risk of contamination and identify potential issues. Alerts are used to monitor for potential dangers, like a patient’s allergy to a medication. Probes are used to monitor for potential errors, like a medication being given at the wrong time or to the wrong patient. Alerts and probes can be particularly helpful when used to monitor antimicrobial use. For example, they can help to identify patients who require antibiotics but aren’t receiving them. They can also help to determine when a specific antibiotic is being used too often, meaning steps can be taken to reduce the use of that drug [22,23].

Alerts and probes are used in combination with medication administration records (MARs). Alerts and probes help you quickly see where potential issues may be occurring. Alerts allow you to take action if necessary; they don’t report errors or problems on their own. When used in combination with MARs, alerts can help reduce the risk of contamination and prevent errors from occurring during medication preparation, delivery, and administration [24]. 

The World Health Organization (WHO) created Digital Accelerator Kits (DAKs) to convert narrative guidance into a standardized format that is more easily digitalized and included in decision support systems. DAKs are the standardized documentation of the fundamental aspects of digital client records, such as common workflows, fundamental data elements, scheduling logic and decision-support algorithms, metrics, and reporting indications [25]. It is also possible to utilize a clinical decision support tool that combines an alert from an electronic medical record with a navigator for antimicrobial stewardship [26].

Using Computerized Medication Records

Computerized medication records (CMARs) also play a key role in preventing errors related to medications. In addition to providing complete and accurate information about each patient’s medications on one page, CMAR permits automatic counting of doses delivered or remaining at the end of each shift or day (dehydration reports). CMAR computers can also produce an inventory report indicating the number of units left at any time during an assignment, so nurses know whether they have available stocks on hand. Computerized physician order entry ensures that the order is legible and comprehensive, with all required details such as dose, route, and dosage form included. It also calculates dosage adjustments depending on clinical factors like weight or renal function [27].

Educating Staff

The importance of multi-professional education and training in facilitating optimal clinical practice has been recognized. Effective antimicrobial stewardship involves more than just limiting the use of antibiotics. It also involves educating staff regarding alternative treatment methods - like how to use gloves and hand sanitizer to prevent the transmission of infections. It also involves educating staff about the transmission of infections in general. This includes teaching them how to avoid the transmission of infections from one patient to another. It also includes teaching them how to prevent the transmission of infections from patients to staff and vice versa [28]. Educating staff about infection control and antibiotic stewardship will go a long way towards preventing the spread of drug-resistant bacteria in a healthcare setting.

This enhances healthcare quality by providing effective treatment when it's needed and preventing overuse of antibiotics - which leads to resistance - in other patients who don't really need them [11].

Ensuring that Only the Right Drugs Are Used at the Right Time

Antimicrobial resistance can be curtailed by ensuring that only the right drugs are used at the right time. “Five rights of medication: the right patient, the right drug, the right time, the right dose, and the right route” can be implemented to ensure the right medicine to the right patient [29]. This may mean changing your organization’s policies as far as when certain antibiotics are prescribed [30]. It may also mean changing the policies as far as when certain antibiotics are used for prevention. Ensuring that only the right drugs are used at the right time can reduce the overall amount of antibiotics used in the healthcare setting [31].

Creating a culture of prevention

Antimicrobial stewardship is all about preventing the spread of drug-resistant bacteria in a healthcare setting. And the best way to do that is by encouraging a culture of prevention among patients and staff. To do this, healthy behaviors can be promoted - like washing your hands, eating a healthy diet, and getting enough sleep [32]. Also, techniques like isolation and barrier precautions like the use of Personal Protective Equipment (PPE), transportation of waste, respiratory hygiene, and cleaning and disinfection can be promoted to prevent the transmission of infections [33].

Organizational changes required for effective stewardship

To implement effective antimicrobial stewardship practices, organizations will likely need to make a few organizational changes. This includes gathering data from organizational surveillance to determine the source of infections and implementing policies to reduce the risk of infection. Organizing information about infections to help identify where they’re coming from is important because it helps you understand where you need to focus your efforts. This information can be gathered by instituting a standardized infection-reporting system. Organizations can also enlist the help of pharmacists, microbiologists, and physicians to help interview patients and look into the medical records about recent infections [34,35].

Limiting the Use of Antibiotics as a Prevention Strategy

Antibiotics are primarily used to treat infections - not to prevent them. But there are certain situations where antibiotics are used for prophylaxis - that is, to prevent infections from occurring in the first place. To prevent infections from occurring, healthcare facilities might prescribe antibiotics for: - Patients who are at a high risk of contracting an infection - Patients who are being treated for an infection but are at a high risk of contracting a different infection - Patients who are at elevated risk of contracting an infection because of a health condition - Patients who are at a high risk of developing a healthcare-associated infection - Patients who are undergoing invasive procedures - Patients who are receiving chemotherapy treatment [36].

Some bacteria - particularly gram-negative pathogens like *Klebsiella pneumoniae* or *Escherichia coli *- need only a small change in their genetic makeup to become antibiotic-resistant [37]. One of the issues with antibiotic resistance is that it allows healthcare-associated infections to become more difficult and expensive to treat than they otherwise would have been. Another issue with antibiotic resistance is that it allows some bacterial infections to spread more readily in hospitals, making them extremely difficult to control. Because of these issues, many institutions have started using antibiotics only as a last resort and not as an initial strategy for preventing infections - especially because there are other infection control practices such as hand hygiene and isolation strategies that can help prevent infections from happening in the first place.

Using Cleaner Environments With Better Airflow

Infections are more likely to occur in areas where there are poor environmental conditions - such as rundown buildings, storage facilities, and laundry rooms [38]. Safe water consumption, appropriate containment, treatment, and disposal of human waste and other wastewater, including that from healthcare facilities, as well as good personal hygiene habits, proper disposal of solid waste, including unused and expired medicines is required to prevent the spread of resistant microorganisms and the development of diseases that may require antibiotic treatment [39]. Because of this, many institutions have started paying close attention to how clean their facilities and equipment actually are.

Health care facilities should have a defined antimicrobial stewardship policy in place that is available to all stakeholders. The policy should be evidence-based, regularly updated, and communicated clearly both verbally and through visual means such as posters. All staff should be trained on the proper use of antimicrobials as well as how to report misuse.

## Conclusions

Antimicrobial stewardship is an important practice for preventing the spread of drug-resistant bacteria in healthcare settings. There are several ways that antimicrobial stewardship practices can be implemented in the healthcare setting, including limiting the use of antibiotics and promoting healthy behaviors. With these strategies in place, infections can be prevented from occurring in the first place - meaning, you’ll need fewer antibiotics overall. This will help reduce the risk of antibiotic resistance and ensure that patients receive the care they need when they need it - even in the face of an antibiotic-resistant infection.
